# The effects of socio-economic status and physical health on the long-term care needs of Japanese urban elderly: a chronological study

**DOI:** 10.1007/s12199-012-0287-5

**Published:** 2012-07-03

**Authors:** Suwen Yang, Tanji Hoshi, Naoko Nakayama, Shuo Wang, Fanlei Kong

**Affiliations:** Graduate School of Urban Environmental Science, Tokyo Metropolitan University, 1-1 Minami-Osawa, Hachioji, Tokyo, 192-0397 Japan

**Keywords:** Long-term care needs, Socio-economic status, Physical health, Urban elderly, Structural causal relationship

## Abstract

**Objectives:**

The aim of this chronological study was to elucidate the effects of socio-economic status (SES) and physical health on the long-term care (LTC) needs of a Japanese elderly population and to explore their causal relationships.

**Methods:**

A self-administered questionnaire was distributed to all residents aged 65 years and older of Tama City, Tokyo, in September 2001. A total of 13,195 completed questionnaires were returned, giving a response rate of 80.2 %. A follow-up study was done using the same questionnaire in 2004. Ultimately, 7,905 respondents were included in our analysis. Data analysis was performed using correlation analysis and structural equation modeling (SEM). For SEM, we used one observed variable (LTC needs in 2004) and three latent variables (SES in 2001 and physical health in both 2001 and 2004).

**Results:**

The data were well fit by the models, with a NFI of 0.980, CFI of 0.982, and RMSEA of 0.032. LTC needs were well explained by the three latent variables (*R* ^2^ = 0.70 and 0.66 for elderly men and women, respectively). Among all variables, physical health in 2004 was the strongest determinant of LTC needs, followed by physical health in 2001, and SES in 2001. Gender differences in the structural relationships were minor.

**Conclusions:**

Our results indicate that good physical health directly contributes to reducing LTC needs among Japanese elderly. In addition, efforts to increase income and educational levels may help to decrease LTC needs by indirectly improving physical health.

## Introduction

The size and proportion of the aging population is increasing worldwide due to increasing longevity and declining birth rates. Japan is the most rapidly aging society in the world. In 1990, elderly people (≥65 years old) represented 12 % of the Japanese general population and this figure is projected to be around 40 % in 2050 [1]. Japan currently also has the longest life expectancy at birth (79 years for males and 86 years for females in 2010). The increase in the numbers of this aging population has increased the need for more affordable health and welfare services for the elderly and is challenging the sustainability of social security systems in Japan.

In 2000, the Japanese government implemented public long-term care (LTC) insurance available to those aged 65 years and older who require sustained nursing care and those aged 40–64 years with one of 15 specified diseases are eligible. During the 10-year period since the implementation of this program (up to April 2010), the number of people certified as requiring LTC has increased by about 2.69 million (123 %) [2, 3].

An analysis of national survey data before and after the program was initiated showed an increased use of formal care at lower costs to households, but mixed results for its effects on personal careers [4]. Evidence from micro-level household data suggests that introducing the LTC system helped Japanese households to reduce income losses associated with a disabled family member [5]. However, the increased demand for LTC will result in an increased cost to society, which raises concerns regarding the control of health-related expenditures. Therefore, it is necessary to explore the factors that are associated with this increased demand for LTC.

In the LTC field, there is a consensus that disability among the elderly is the main factor driving the demand for LTC services [6]. Using the ratio of total number of elderly certified for LTC support/care versus total elderly population as a measure of disability prevalence, the disability status of the elderly population in Japan was 9.9 % in 2000 versus 16.3 % by 2006 [7].

Although numerous studies have been made on the variables associated with increased health care needs and health expenditures, such as income, family context and health-related factors, little is known of the factors that are related to increased LTC needs. More specifically, little is known regarding the underlying mechanisms or processes of each factor.

The purpose of this chronological study was to elucidate the effects of socio-economic status (SES) and physical health on LTC needs among the urban elderly in Japan. Three hypotheses for LTC needs were examined: (1) SES would affect LTC needs either directly or indirectly; (2) physical health, as determined by SES, would directly affect LTC needs; (3) there are gender differences in LTC needs and the factors that influence these needs. Finally, a structural model explaining the causal relationships between LTC needs and other factors that influence it was developed and validated.

## Methods

### Study objects

In September 2001, a questionnaire survey of all urban elderly (aged ≥65 years) residents of Tama city, Tokyo, was conducted. Of the 16,462 eligible elderly who received a questionnaire, 13,195 returned the completed questionnaire (respondents), which was a response rate of 80.2 %. A follow-up survey using the same questionnaire was conducted in September 2004, and 8,558 participants responded (505 had moved, 914 had died, and 3,218 did not respond). Our final sample was therefore limited to 7,905 respondents for whom we had valid observations for the variable associated with LTC needs (Table 1).

**Table 1:** Descriptive characteristics of the study subjects

Study subjects	LTC needs in 2004						χ^2^ test
	No LTC needs (*n* = 7,366)		LTC needs (*n* = 539)		Total (*n* = 7,905)		
	*n*	%	*n*	%	*n*	%	
Gender							
Male	3,563	48.4	201	37.3	3,764	47.6	*p* < 0.001
Female	3,803	51.6	338	62.7	4,141	52.4	
Age							
65–74 years (younger elderly)	4,411	59.9	162	30.1	4,573	57.8	*p* < 0.001
≥75 years (older elderly)	2,955	40.1	377	69.9	3,332	42.2	
BADL score 2001							
0	4	0.1	29	5.4	33	0.4	*p* < 0.001
1	5	0.1	19	3.5	24	0.3	
2	578	7.8	95	17.6	673	8.5	
3	6,618	89.8	374	69.4	6,992	88.5	
Missing	161	2.2	22	4.1	183	2.3	
IADL score 2001							
0	21	0.3	64	11.9	85	1.1	*p* < 0.001
1	36	0.5	57	10.6	93	1.2	
2	55	0.7	55	10.2	110	1.4	
3	115	1.6	41	7.6	156	2.0	
4	547	7.4	65	12.1	612	7.7	
5	6,419	87.1	216	40.1	6,635	83.9	
Missing	173	2.3	41	7.6	214	2.7	
Frequency of going outside 2001							
<Once a month	203	2.8	92	17.1	295	3.7	*p* < 0.001
>Once a month	451	6.1	83	15.4	534	6.8	
>3–4 times a week	6,408	87.0	309	57.3	6,717	85.0	
Missing	304	4.1	55	10.2	359	4.5	
BADL score 2004							
0	5	0.1	82	15.2	87	1.1	*p* < 0.001
1	29	0.4	112	20.8	141	1.8	
2	810	11.0	193	35.8	1,003	12.7	
3	6,110	82.9	90	16.7	6,200	78.4	
Missing	412	5.6	62	11.5	474	6.0	
IADL score 2004							
0	35	0.5	120	22.3	155	2.0	*p* < 0.001
1	48	0.7	93	17.3	141	1.8	
2	74	1.0	63	11.7	137	1.7	
3	188	2.6	41	7.6	229	2.9	
4	624	8.5	44	8.2	668	8.5	
5	5,922	80.4	93	17.3	6,015	76.1	
Missing	475	6.4	85	15.8	560	7.1	
Frequency of going outside 2004							
<Once a month	88	1.2	68	12.6	156	2.0	*p* < 0.001
>Once a month	1,055	14.3	186	34.5	1,241	15.7	
>3–4 times a week	5,870	79.7	205	38.0	6,075	76.9	
Missing	353	4.8	80	14.8	433	5.5	
Educational level 2001							
Graduation from junior high school	2,580	35.0	268	49.7	2,848	36.0	*p* < 0.001
Graduation from high school	2,644	35.9	123	22.8	2,767	35.0	
Graduation from junior college or higher	1,621	22.0	87	16.1	1,708	21.6	
Missing	521	7.1	61	11.3	582	7.4	
Annual income 2001							
<$13,000	419	5.7	58	10.8	477	6.0	*p* < 0.001
<$39,000	2,520	34.2	230	42.7	2,750	34.8	
<$91,000	2,991	40.6	140	26.0	3,131	39.6	
>$91,000	582	7.9	23	4.3	605	7.7	
Missing	854	11.6	88	16.3	942	11.9	

*LTC* long-term care,* BADL* basic activities of daily living,* IADL* instrumental activities of daily living

All participants provided written informed consents along with their mailed-in questionnaires. The authors signed an agreement with municipal authorities in which they pledged to protect the confidentiality of participants’ private information. In addition, the study protocol was approved by the ethics committee of the respective institutions on September 16, 2004.

### Measures

#### LTC needs

The category certification of LTC was evaluated according to the six levels designated by the Japanese Ministry of Health, Labor and Welfare, which include one support level and five care levels. In our analysis, we only considered the LTC needs in 2004 as the endogenous variable. A respondent that did not receive LTC scored 0, while a respondent scored 1 if assigned the lightest support level and 6 if assigned the most severe care level. All subjects were divided into two groups: “no-LTC needs” and “LTC needs” (Table 1).

#### Physical health indicators

Three indicators of physical health were used in the questionnaire surveys conducted in 2001 and 2004: basic activities of daily living (BADL); instrumental activities of daily living (IADL); frequency of going outside (Table 1). 

The BADL score was determined with three items from the Katz Index (going to the toilet; taking a bath; taking a walk outside) that were rated on a 2-point response scale: 0, requiring assistance; 1, without assistance [8]. BADL scores ranged from 0 to 3 points, with a higher score indicating a greater level of basic activity competence.

The IADL score was based on five activities: (1) buy daily necessities; (2) cook daily meals; (3) conduct banking transactions; (4) manage insurance and pension; (5) read newspapers and books [9]. IADL were rated in the same way as BADL, with scores ranging from 0 to 5 points (higher scores indicated superior levels of instrumental activity).

The frequency of going outside was evaluated from answers to the question, “how many times do you go outside?” This was rated on a 3-point scale (1, less than once a month; 2, at least once a month; 3, at least 3–4 times a week). If the answer was unknown, a score of zero was assigned.

Cronbach’s alpha coefficient for the reliability of these three items was 0.75, indicating they had internal consistency for this investigation.

#### Socio-demographic indicators

We obtained socio-demographic information on our subjects, including gender, age, educational level, and annual income. The subjects were divided into two age groups: “younger elderly” (aged 65–74 years) and “older elderly” (aged ≥75 years). Educational level was a three-level ordinal variable (1, graduation from junior high school; 2, graduation from high school; 3, graduation from junior college or higher). The respondents chose one of the four categories that best corresponded to their annual income (1 = <$13,000; 2 = <$39,000; 3 = <$91,000; 4 = >$91,000; $1.00 ≈ 77 yen). If the answer was unknown, a score of zero was assigned.

### Statistical analysis

Two analysis levels were performed for this study. The first was a correlation analysis that used the χ^2^-test to determine whether the two groups of LTC needs were distributed differently among the categories. This analysis was performed using SPSS ver. 17.0 software for Windows (SPSS, Chicago, IL).

The second analysis involved structural equation modeling (SEM) to explore the underlying causal structure of the relationships among each factor. This analysis was performed using AMOS ver 17.0 software for Windows (SPSS). For the SEM, we used one observed variable (LTC needs in 2004) and three latent variables (SES in 2001 and physical health in both 2001 and 2004). Estimation of the best-fitting model was carried out by the method of maximum likelihood. The optimization algorithm was implemented with no-missing-data parameters. In addition, the direct, indirect, and total effects of each latent variable on the endogenous variable were determined by subject gender. Group comparison analysis was done to examine gender differences/similarities in the measurement and structural relationships [9]. The statistics used for goodness of fit were chi-square, Normalized Fit Index (NFI), Comparative Fit Index (CFI), and root mean square error of approximation (RMSEA). A model was considered to have a good fit when the NFI and CFI were >0.90 and RMSEA was <0.05. A *p* value below 0.05 was considered to be statistically significant.

## Results

### Descriptive analysis

Data were available for a total of 7,905 subjects, of whom 7,366 were in the no-LTC needs group and 539 were in the LTC needs group in 2004 (Table 1).

There was no difference in terms of gender in the no-LTC needs group, while there were nearly twice as many females (62.7 %) as males (37.3 %) in the LTC needs group. In the two age groups, there were more younger elderly (59.9 %) than older elderly (40.1 %) in the no-LTC needs group, while there were nearly twice as many older elderly (69.9 %) than younger elderly (30.1 %) in the LTC needs group.

Although most of the subjects had high BADL and IADL scores in both 2001 and 2004, we did find the following differences. In terms of BADL score, 89.8 % of the elderly who did not need LTC and 69.4 % of the elderly who did need LTC scored 3 points for BADL in 2001, indicating that most of the elderly could take care of themselves. However, these proportions had decreased 3 years later; only 16.7 % of the elderly scored 3 points for BADL in the LTC needs group in 2004.

In terms of IADL score, 87.1 % of the elderly who did not need LTC and only 40.1 % of the elderly who did need LTC scored 5 IADL points in 2001; however, 3 years later, the largest proportion of the elderly in the LTC needs group scored (22.3 %) scored 0 points for IADL.

The frequency of going outside showed the same patterns as both the BADL and IADL scores. Compared to the no-LTC needs group, fewer elderly in the LTC needs group were able to go outside at least 3–4 times per week in both 2001 and 2004. However, the frequency of going outside declined from 2001 to 2004 for all subjects.

Compared to the LTC needs group, those in the no-LTC needs group had attained higher educational levels and earned much more money in 2001. There were many more elderly in the no-LTC needs group than in the LTC needs group who had graduated from high school and college (57.9 vs. 38.9 %) and who earned more than $91,000 (48.5 vs. 30.3 %).

There were significant statistical differences between the no-LTC needs group and the LTC needs group for all variables by the χ^2^ test.

### Structural equation analysis

**Fig. 1:**
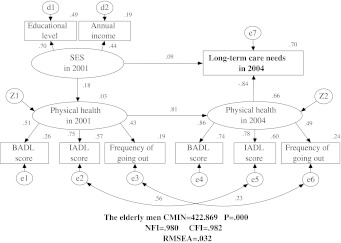
Causal structure of long-term care needs with socio-economic status (*SES*) and physical health for the elderly men. There are nine observed endogenous variables [educational level, annual income, basic activities of daily living (*BADL*) score in 2004, instrumental activities of daily living (*IADL*) score in 2001, frequency of going out in 2001, BADL score in 2004, IADL score in 2004, frequency of going out in 2004, and long-term care needs in 2004], two unobserved endogenous variables (physical healthy in 2001 and physical health in 2004), and 12 unobserved exogenous variables (SES in 2001, d1, d2, z1, z2, e1, e2, e3, e4, e5, e6, e7). *Arrows* indicate their significant associations and their directions between variables, *double curved arrows* indicates correlation between each factor. This model fit the data well with the high goodness of fit indexes which are showed in the figure. The long-term care (LTC) needs of elderly men are well explained by the three latent variables (*R* ^2^ = 0.70).* NFI* Normalized Fit Index,* CFI* Comparative Fit Index,* RMSEA* root mean square error of approximation, *CMIN* chi-square (χ^2^)

**Fig. 2:**
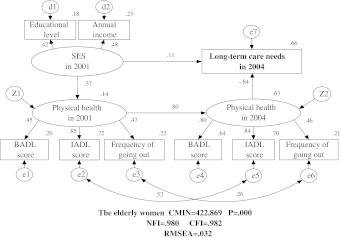
Causal structure of long-term care needs with SES and physical health for the elderly women. There are nine observed endogenous variables (educational level, annual income, BADL score in 2001, IADL score in 2001, frequency of going out in 2001, BADL score in 2004, IADL score in 2004, frequency of going out in 2004, and long-term care needs in 2004), two unobserved endogenous variables (physical healthy in 2001 and physical health in 2004), and 12 unobserved exogenous variables (SES in 2001, d1, d2, z1, z2, e1, e2, e3, e4, e5, e6, e7). *Arrows* indicate their significant associations and their directions between variables, *double curved arrows* correlation between each factor. This model fit the data well with the high goodness of fit indexes which are showed in the figure. The LTC needs of elderly women are well explained by the three latent variables (*R* ^2^ = 0.66)

Figures 1 and 2 show the models for elderly men and elderly women, respectively. The models fit the data very well, with a NFI of 0.980, a CFI of 0.982, and a RMSEA of 0.032. All of the loading included in the models were statistically significant (*p* < 0.001). LTC needs were well explained by the three latent variables included in the models (*R* ^2^ = 0.70 for elderly men and *R* ^2^ = 0.66 for elderly women). These models describe four pathways: (1) starting from the underlying SES in 2001 via physical health both in 2001 and 2004 leading to the endogenous observed LTC needs in 2004; (2) starting from physical health in 2001 via physical health in 2004 leading to LTC needs in 2004; (3) and (4) two direct pathways for SES in 2001 and physical health in 2004 approaching LTC needs in 2004, respectively. However, there was no significant direct relationship between SES in 2001 and physical health in 2004.

Standardized estimates of the different latent variables on LTC needs in 2004 are included in Table 2. Overall, physical health in 2004 was the strongest determinant of LTC needs, followed by physical health in 2001, and SES in 2001. The standardized direct effects of physical health in 2004 on LTC needs were −0.844 for men and −0.839 for women. The indirect effects of physical health in 2001 on LTC needs were also strong, namely, −0.683 for men and −0.668 for women. Compared to the direct effects of SES on LTC needs, the indirect effects of SES were stronger for both men (−0.125 vs. 0.088) and women (−0.249 vs. 0.113).

**Table 2:** Standardized effects of direct, indirect, and total related factors on long-term care needs by gender

Variables	Elderly men			Elderly women		
	Direct	Indirect	Total	Direct	Indirect	Total
Socio-economic status 2001	0.088	−0.125	−0.038	0.113	−0.249	−0.136
Physical health 2001	–	−0.683	−0.683	–	−0.668	−0.668
Physical health 2004	−0.844	–	−0.844	−0.839	–	−0.839

Regarding gender comparisons, the effects of SES on LTC needs were greater for women than for men, and this difference was statistically significant. In contrast, the physical health effects on LTC needs were greater for men than for women. However, the differences were very slight.

## Discussion

The results of our study indicate that SES and physical health were significant predictors of the LTC needs of our study population. Firstly, our results verify a strong causal association between physical health and LTC needs; as such they correspond quite well with actual situations. In Japan and most other developed countries, the demand for LTC services has been found to increase as the number of elderly people with disabilities or requiring support in their ADLs also increases [6, 10]. It is important to note that we found a trend for deteriorating physical health status among all subjects in this follow-up study (Table 1). From 2001 to 2004, the percentage of our elderly population who scored 3 points for BADL decreased from 88.5 to 78.4 %, the percentage who scored 5 points for IADL decreased from 83.9 to 76.1 %, and the percentage of the elderly who went outside at least 3–4 times per week decreased from 85.0 to 76.9 %. Moreover, the standardized indirect effects of physical health in 2001 and the direct effects in 2004 on LTC needs indicated that with improved physical health, the levels of LTC needs would be decreased. Therefore, these results have several policy implications to ensure the sustainability of the LTC system. In order to decrease LTC needs and expenditures in the future, it will be important to improve daily living capabilities and reduce the number of dependent elderly through better prevention because prevention is important not only for averting cost–push pressures on health expenditures, but also for improving peoples’ qualities of life [11].

Secondly, although the direct effect of SES on LTC needs was not strong, the direct effects of SES on physical health in 2001 and an indirect effect on physical health in 2004 were stronger. The impact of SES on physical health has been investigated among the elderly worldwide, and there appears to be a consistent inverse relationship between SES and disability [12–17]. Our findings indicate that any improvement in individual incomes and educational levels will work towards improving daily living capabilities, decreasing disabilities and, consequently, decreasing LTC needs.

Finally, in this study we also focused on a comparison of the genders. In general, the elderly women of our study population had a lower SES and accounted for a higher proportion of people in the LTC needs group than elderly men. Societal stratification seems to be increasing year by year [18, 19]. However, our structural equation analysis results revealed that the gender differences were slight.

In conclusion, our study examined the effects of SES and physical health as possible explanations for LTC needs among elderly Japanese urban dwellers. The results revealed that good physical health or a high ability to perform ADLs directly contributed to reducing LTC needs. In addition, efforts to increase income and educational levels may help to decrease LTC needs via improving physical health indirectly. It will be necessary to perform an interventional study in the future to reduce LTC needs.
